# Greater inequalities in dental caries treatment than in caries experience: a concentration index decomposition approach

**DOI:** 10.1186/s12903-021-01935-z

**Published:** 2021-11-08

**Authors:** Yuandong Qin, Lin Chen, Jianbo Li, Yunyun Wu, Shaohong Huang

**Affiliations:** 1 Stomatological Hospital, Southern Medical University, No. 366, South of Jiangnan Avenue, Guangzhou, China; 2 Chongqing Center for Disease Control and Prevention, The Second Changjiang Road No. 8, Chongqing, 400042 China

**Keywords:** Dental caries, Socioeconomic-related oral health inequality, Concentration index, Welfare policy

## Abstract

**Background:**

The aim of the current study was to (a) measure the socioeconomic inequalities in oral health and examine whether the inequalities are greater in disease experience or in its treatment and to (b) decompose the factors that influence oral health inequalities among the adults of Guangdong Province.

**Methods:**

A cross-sectional study was conducted among 35- to 44-year-old and 65- to 74-year-old adults in Guangdong Province. All participants underwent oral health examinations and answered questionnaires about their oral health. We measured the concentration indices of the DMFT and its separate components, namely, decayed teeth (DT), missing teeth (MT), and filled teeth (FT), to explore the inequalities in oral health status; then, we analysed its decomposition to interpret the factors that influence the inequalities.

**Results:**

The results showed that significant inequality was concentrated on FT (CI =  0.24, 95% CI = 0.14/0.33, SE = 0.05). The concentration indices for the DMFT (CI =  0.02, 95% CI =  0.02/0.06, SE = 0.02) and MT (CI =  0.02, 95% CI 0.03/0.08, SE = 0.03) were small and close to zero, while the concentration for DT (CI =  − 0.04, 95% CI =  − 0.01/0.02, SE = 0.03) was not statistically significant. The results from the decomposition analysis suggested that a substantial proportion of the inequality was explained by household income, high education level, regular oral examination and type of insurance (5.1%, 12.4%, 43.2%, − 39.6% (Urban Employee Basic Medical Insurance System) and 34.5% (New-Type Rural Medical Collaboration System), respectively).

**Conclusions:**

The results indicated greater inequalities in dental caries than in caries experience. Among the included factors, household income, high education level, and regular oral health examinations had the greatest impact on the inequalities in the number of FT. In addition, the current medical insurance systems, including the Urban Employee Basic Medical Insurance System, Urban Resident Basic Medical Insurance System, and the New-Type Rural Medical Collaboration System, have not been effectively used in oral treatment. Policy-making and the implementation of interventions for tackling socioeconomic oral health inequalities should focus on reducing the burden of treatment and providing greater access to dental care for low-income groups. Welfare policies are skewed towards rural areas and low-income people.

## Background

Socioeconomic-related oral health inequalities are a major concern [1] worldwide and exist in both developed and developing countries [2–5]. A study from the United States [6] reported that periodontal disease inequalities are pervasive and associated with education, income and race/ethnicity. In the United Kingdom [7], social gradients in regard to dental caries are common in both children and adults. Socioeconomic disparities in oral health care are both unnecessary and avoidable, and even more important, they are considered unfair and unjust [8]. Therefore, socioeconomic inequalities in oral health have drawn extensive attention. For instance, the international Centre for Oral Health Inequalities Research and Policy (ICOHIRP) was founded in 2013, and the London Charter on Oral Health Inequalities was subsequently published in 2015 [4].

With a permanent resident population of 108.49 million in 2015 (Data from STATS.GD.GOV. CN: http://stats.gd.gov.cn/gdtjnj/content/post_1424895.html), Guangdong Province is located in southern China and covers an area of 174,246 km^2^ (Data from Ministry of Civil Affairs of the People’s Republic of China: http://xzqh.mca.gov.cn/defaultQuery?shengji=%B9%E3%B6%AB%CA%A1%28%D4%C1%29&diji=-1&xianji=-1). Since 1989, the economic aggregate of Guangdong has ranked first among the 31 provinces of China (Data from National Bureau of Statistics: http://www.stats.gov.cn/tjsj/ndsj/2015/indexch.htm). The province suffers from inequalities in the economic development level [9], and the socioeconomic gradient is closely associated with oral disease distribution [10–12]. As a developed region in China, the current situation of Guangdong Province can predict the future development of other regions. However, there is a lack of research on socioeconomic-related oral health inequalities in this region of China. This situation is not conducive to the development of policies to promote oral health equality.

Socioeconomic inequalities in oral health have been reported both internationally and nationally to explore measures to reduce oral health inequalities. Tselmuun Chinzorig [13] reported inequalities in caries experience among Mongolian children. Mengru Xu [14] measured and decomposed socioeconomic-related inequality in the use of oral health services among Chinese adults and found that oral health care service utilization was disproportionately concentrated among better-off Chinese adults. These studies focused either on a single aspect of oral disease or on oral health care service utilization. However, oral disease and oral health care services together affect oral health outcomes, which should be considered in research on oral health inequalities. Dental caries are a long-term chronic disease whose lifelong effects and management can be directly observed and measured. Dental caries are commonly measured by the DMFT index and its components, namely, DT (decayed teeth), MT (missing teeth), and FT (filled teeth). The DMFT is the sum of DT, MT and FT, which reflects one’s actual disease experience (past and present), while DT, MT, FT separately indicate the management of that disease [15]. To address social inequalities in oral health, it is very important to understand whether oral health inequalities are due to the experience of disease or to the treatment of disease.

Previous studies have usually assessed oral health inequality through regression-based methods [16] or variation in mean health across quintiles of the oral health index [17]. Although convenient and easy to interpret, such a grouped analysis provides only a partial picture of how health varies across the full distribution of the oral health index. A complete picture can be provided using a concentration index, which displays the share of health accounted for by cumulative proportions of individuals in a population ranked from poorest to richest [17]. Moreover, the decomposition of the concentration index helps to analyse which variables contribute to the inequality [17]. In this context, the use of the concentration index and its decomposition approach has permitted an understanding of contributors to socioeconomic inequalities in oral health.

The aim of this study was to explore the socioeconomic inequalities of oral health in Guangdong Province that exist mainly in disease experience or treatment and to analyse the factors that influence the inequality by a concentration index decomposition approach.

## Methods

### Study design and sample

This study presents an analysis of the data from an epidemiological survey of oral health status in Guangdong Province conducted from December 2015 to April 2016.

A cross-sectional study was conducted among two age groups (35–44-year-olds and 65–74-year-olds) of adults in Guangdong Province. In this study, we obtained a representative sample by using a multistage stratified cluster sampling method with selection probabilities proportional to size (PPS) [18]. In the first stage, four districts and four counties were chosen randomly by stratified sampling using the probability-proportional-to-size (PPS) method. In the second stage, the PPS method was used to select three neighbourhood committees in each district and three village committees in each county. In the third stage, the individuals who were interviewed in the selected communities were chosen using a quota sampling method [19] that excluded people with serious physical or psychological illness or disadvantages, as well as those who were unable or unwilling to finish the survey. The sample size for each age group was estimated from the following formula:

$$N = {\text{deff}}(\mu^{2} p(1 - p){/}\delta^{2}$$

N represents the sample size. Considering the stratification of urban–rural and male–female, the sampling design efficiency ‘deff’ was set to 3.0. Furthermore, p represents the prevalence of caries among 35–44-year-old adults of Guangdong in the Third National Oral Health Survey [20], which was 63.9%. The level of confidence is μ = 1.96, and the margin of error is δ = 10%. The required sample size for each age group was 266 according to the formula. Finally, 576 participants responded to the invitation and completed the survey, with 288 participants in each age group.

This study was approved by the Stomatological Ethics Committee of the Chinese Stomatological Association on July 9, 2014 (Approval No.: 2014-003). Before the study, all the participants were provided with all the details of the survey, and signed informed consent was obtained.

### Data collection

Data were collected through structured questionnaires and oral examinations at the corresponding communities. The structured questionnaire [21, 22] solicited information on household income, education, oral health knowledge, attitudes, oral health practices and key sociodemographic variables. Prior to the data collection, the questions were pre-tested in comparable groups of adults in order to assess reliability and validity. A sub-sample of participants who were given the same questionnaire 20–30 days after completion of the initial questionnaire and consistency rates of at least 70% were achieved [23]. Trained dentists completed the questionnaires in one-to-one interviews in Chinese, which helped overcome barriers of illiteracy. Furthermore, in districts where dialects were difficult to understand, a local person acted as an assistant. The training sessions for the dentists were held by the Chinese Stomatological Association and Peking University Hospital of Stomatology in Beijing before the start of the survey. Survey staffs were trained by a standard survey staff on the questionnaire content, questionnaire methods, questionnaire skills, details to be paid attention to. After the training, questionnaire consistency assessment was carried out from a sub-sample of participants who completed the questionnaire supervised by the same survey staff 20–30 days after completion of the initial questionnaire, and the consistency rates were more than 90%. The oral examination of the participant’s dental caries status was performed with the aid of a mirror and a ball-ended WHO Community Periodontal Index probe by three calibrated and accredited dentists with the assistance of trained recorders, according to World Health Organization (WHO) criteria [24]. The calibration results were 0.83–0.92, as calculated by kappa statistics. The numbers of decayed teeth and missing teeth and filled teeth were recorded based on the criteria recommended by the WHO [24].

#### Variables

The variables in the study included the following.

##### Oral health outcomes

The participants’ dental caries status was evaluated by analysing the DMFT index, which is commonly used for epidemiological studies in dental research. Teeth were classified as decayed (DT) if there was evidence of cavitation on the crown or root. Teeth do not present for any reason in people 30 years and older were classified as missing (MT). Filled teeth without secondary caries were classified as filled (FT). The total number of DTs, MTs and FTs was recorded as the DMFT score, which was used to assess the participant’s dental caries disease experience [25].

##### Household income

Annual household income was obtained by the question: “What was your approximate total household income in the past 12 months?” Participants were asked to answer with an exact number using the integer value of ¥10,000. To facilitate statistics, we categorized household income as follows: low (≤ ¥20 000/year, approximately US$2903.5/year), medium (¥30 000/year-¥40,000/year, approximately US$4353.8/year–US$5805.0/year), medium–high (¥50 000/year − ¥70,000/year, approximately US$7256.3/year − US$10,158.8/year) and high (≥ ¥80 000/year, approximately US$11,610.0/year) [26, 27] when bivariate analysis and multivariate analysis were performed. The participants were allowed to leave this question unanswered because income is a sensitive issue.

##### Education

Education was classified as no formal schooling, primary school, middle school, high school, technical secondary school, junior college, university completed, or postgraduate degree or above.

##### Health-related behaviour variables

These variables included the consumption of desserts (Yes/No), consumption of sugared drinks (Yes/No), tooth brushing (Yes/No), toothpick use (Yes/No), floss use (Yes/No), last time of dental attendance (never visit dentist, visited dentist 1 year ago, visited dentist 6–12 month ago, visited dentist within last 6 months).

##### Oral health knowledge

Oral health knowledge was measured by eight questions [23], as shown in Table 1, and the quality of measurement is shown in Table 2. The correct answer for each question was coded as 1, and an incorrect answer or “don’t know” answers were coded as 0. The sum of the eight answers created a single oral health knowledge variable ranging from 0 to 8. A score less than or equal to 4 was categorized as "low", and a score greater than 4 was categorized as "high".

**Table 1:** Questionnaire about oral health knowledge and oral health attitudes

Number	Question	Answer
*Questions about oral health knowledge*		
1	Gingival bleeding is normal when brushing teeth	No () Yes () Don’t know ()
2	Germs are one of the reasons for gingivitis	No () Yes () Don’t know ()
3	Toothbrushing is useless to prevent gingivitis	No () Yes () Don’t know ()
4	Dental caries are mainly caused by germs	No () Yes () Don’t know ()
5	Sugar consumption can lead to dental caries	No () Yes () Don’t know ()
6	Fluoride is useless in protecting teeth	No () Yes () Don’t know ()
7	Pit and fissure sealant can protect teeth	No () Yes () Don’t know ()
8	Oral disease can influence systemic health	No () Yes () Don’t know ()
*Questions about oral health attitudes*		
1	My oral health is very important to me	Agree () Disagree () Neither ()
2	Regular dental check-ups are important	Agree () Disagree () Neither ()
3	Tooth condition is decided at birth and is not related to self-care	Agree () Disagree () Neither ()
4	Self-care is important in preventing dental problems	Agree () Disagree () Neither ()

**Table 2:** Reliability and validity of knowledge and attitude

Measure	Cronbach alpha	KMO	*P* of Bartlett Test
Knowledge	0.76	0.78	< 0.001
Attitude	0.73	0.77	< 0.001

##### Oral health attitudes

Four questions [23], as shown in Table 1, were included as items in the oral health attitude summary score, and the quality of measurement is shown in Table 2; the answer for each question was “agree”, “disagree” or “neither”. Responses were coded 1 for a positive attitude and 0 for a negative attitude or neither. The final oral health attitude scores ranged from 0 to 4; scores were categorized as "low" (0–2) and "high" (3–4).

##### Subjective health conditions

These included self-rated oral health (good, fair or poor), chronic disease (yes/no).

##### Socio-demographic variables

These included gender (male or female), age, type of household (non-agricultural family or agricultural family).

##### Insurance information variable

There are three medical insurance systems for citizens in China: the urban employee basic medical insurance for the urban employed, the new-type rural collaboration medical system for rural residents, and the urban resident basic medical insurance covering urban residents without formal employment. Citizens are insured on a voluntary basis. We obtained the insurance information variable by asking the following questions:

Reimbursement for dental treatment: "Do you get reimbursement for dental treatment?" "Yes/No".

Urban Employee Basic Medical Insurance System: "Do you have the urban employee basic medical insurance system?" "Yes/No".

Urban Resident Basic Medical Insurance System: "Do you have the urban resident medical insurance system?" "Yes/No".

The New-Type Rural Medical Collaboration System: "Do you have the new-type rural medical collaboration system?" "Yes/No".

### Analysis

#### Concentration Index

Statistical analyses were carried out adjusted for the complex sample. The sample weights of each sampling stage were the inverse of the probability of selection. The sample weights of each evaluated case were the product of the sample weights of each stage.

Inequalities in oral health status, including the DMFT, DT, MT, and FT, were identified by the concentration index (CI) in the study. The concentration index provides a way to assess the degree of health-related economic inequality [28–30] and is increasingly used in the dental literature [31]. The CI is constructed by a concentration curve (CC). The CC illustrates the distribution of a health variable (Y axis) against an economic variable (X axis). The economic variable (household income) is cumulatively ranked and ranges from the poorest person/household to the richest. In fact, the curve shows among which economic groups health is mostly concentrated. If health is equally distributed across the economic groups, the curve will be a 45-degree line called the ‘equality line’. Otherwise, the curve will lie above or below the equality line, thereby showing the existence of inequality in the distribution of health. The CI value is the area between equality line and the CC. In the case of equality, the CC and the equality line coincide, and the CI is zero. If the CC lies above the equality line, this indicates that health is highly concentrated among people of lower economic status, and the CI will take a negative value. If the CC lies below the equality line, this indicates that health is highly concentrated among people of higher economic status, and the CI will take a positive value [29]. The CI can be represented by following [32, 33]:

$${\text{CI}} = {2/}\mu {\text{ cov(y}}_{i} {\text{,R}}_{i} )$$

where y_*i*_ and R_*i*_ are, respectively, the health status of the *i*th individual and the fractional rank of the *i*th individual in terms of the index of household economic status; μ is the mean of the health of the sample; and cov denotes the covariance.

#### Decomposition of the concentration index

After measurement of the CI, we can go further and decompose the CI to understand which variables contribute to the inequality [30]. To do so, a negative binomial model was chosen since count data showed signs of overdispersion [34, 35]. Wagstaff and colleagues [17] wrote a nonlinear model of the relationship between a health variable, y, which may be count data, and factors (x) in terms of a general functional form G:

a $$y_{i} = G\left( {\alpha + \mathop \sum \limits_{i} \beta_{i} x_{i} } \right) + \varepsilon_{i}$$

where G takes a particular form for the negative binomial model. The concentration index for y, CI can be written as follows:

b $$CI = \mathop \sum \limits_{K} (\beta_{k} \overline{x}_{k} /\mu )C_{k} + GC_{\varepsilon } /\mu$$

where μ is the mean of y, $\overline{x}_{k}$ is the mean of $x_{k}$, C_k_ is the concentration index for $x_{k}$, and GCε is the generalized concentration index for the error term (ε).

Equation (b) consists of two components: (1) an explained component and (2) an unexplained component. The first component is made up of two constituents: elasticity and the CI of regressors. The second component, the unexplained part, is the part of the inequality that cannot be explained by systematic variation in the determinants across economic groups. To perform the decomposition, the values of all variables in Eq. (b) should be calculated. First, the coefficients (β_k_) of the explanatory variables are calculated. To do so, a regression analysis using an appropriate regression model must be conducted. In the present study, considering that FT is a continuous variable and not normally distributed, nonparametric tests, namely, the Mann–Whitney U-test and Kruskal–Wallis H(K) test, were used to evaluate the bivariate associations between each explanatory variable and FT. Then, linear regression was used to calculate the coefficient of explanatory variables. In the second step, the means of the health variable (μ) and each determinant ($\overline{x}_{k}$) are calculated. All the variables in Eqs. (a) and (b) are calculated, and one can reveal the contribution of each determinant to inequality by multiplying the elasticity of each determinant by $\left( {\frac{{\beta_{k} \overline{x}_{k} }}{\mu }} \right)C_{k}$. This is the absolute contribution of each determinant to the measured inequality. In the last step, to calculate the percentage contribution, the absolute contribution of each determinant is divided by the CI of the health variable $\left( {\frac{{\beta_{k} \overline{x}_{k} }}{\mu }} \right)C_{k}$/CI. The contribution of an X variable to the measured health inequality can be either positive or negative. A positive contribution shows that the variable would add to the inequality in the health variable and vice versa.

Statistical analyses were conducted using Stata MP 13. The level of statistical significance for the tests mentioned was set at *p* < 0.05.

## Results

### Sample characteristics

The final sample consisted of 576 participants aged 35–44 years and 65–74 years. The basic characteristics of dental caries in the study participants are shown in Table 3. Nearly all (95.6%) participants reported having dental caries experience (DMFT ≥ 1), and the mean DMFT score was 8.51 (95% CI 7.85/9.18). The prevalence rate for DT ≥ 1 was 66.1%, and the mean number of DTs was 2.30 (95% CI 1.94/2.66). The mean number of MTs was 5.35 (95% CI 4.68/6.01), and 84.6% of participants suffered from missing teeth. Surprisingly, only 31.1% of participants received dental caries treatment, and the mean number of FTs was 0.87 (95% CI 0.51/1.22).

**Table 3:** Basic characteristics of dental caries in the study participants

	Outcome ≥ 1 (%)	Mean	95% CI	
DMFT	95.6	8.51	7.85	9.18
DT	66.1	2.30	1.94	2.66
MT	84.6	5.35	4.68	6.01
FT	31.1	0.87	0.51	1.22

DMFT, decayed, missing and filled teeth; DT, decayed teeth; MT, missing teeth; FT, filled teeth

### Concentration curve and concentration index

**Fig. 1:**
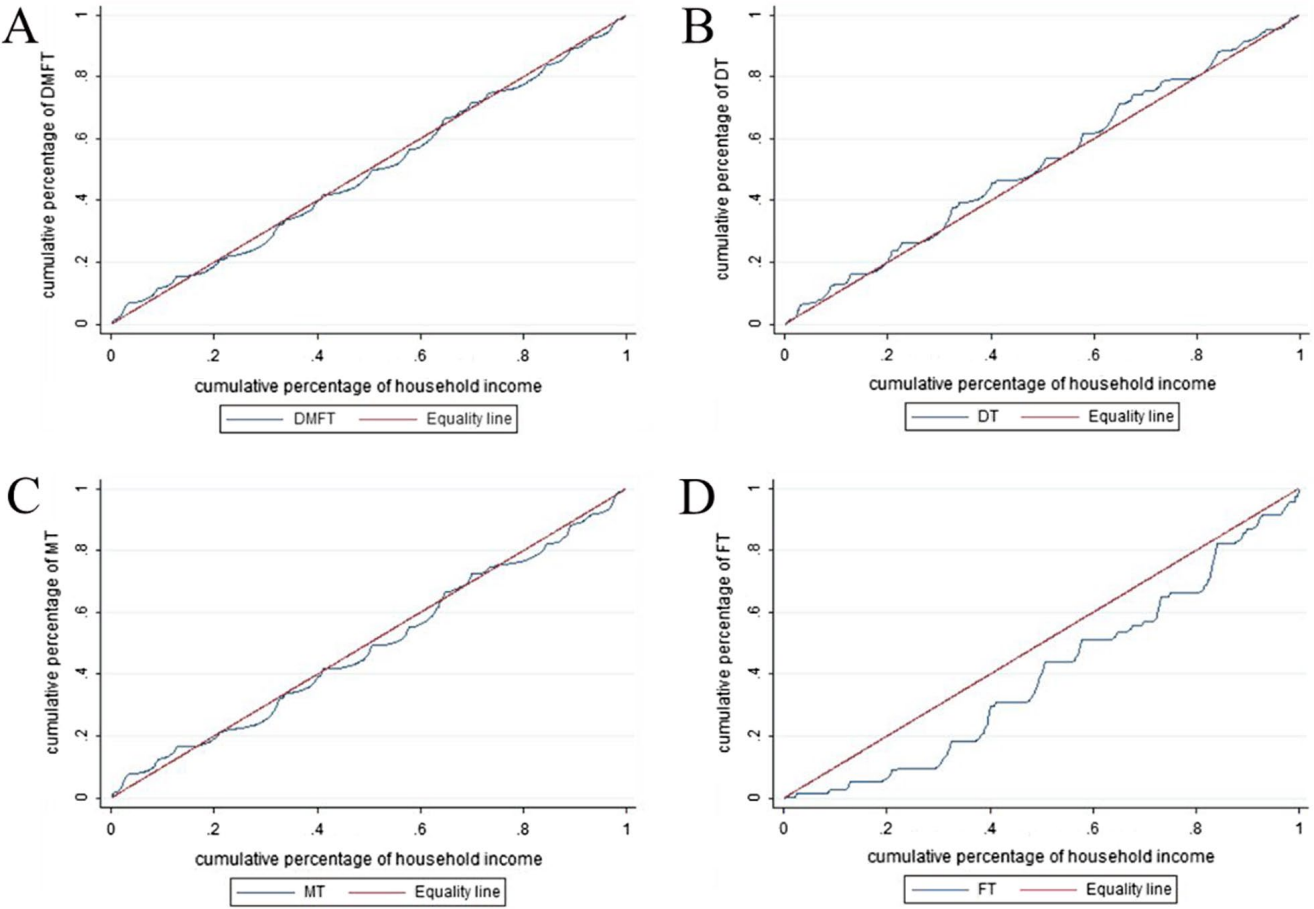
Concentration curve of DMFT, DT, MT and FT. **A** Concentration curve of DFMT. **B** Concentration curve of DT. **C** Concentration curve of MT. **D** Concentration curve of FT

Figure 1 presents the concentration curves for oral health outcomes in the participants, and the corresponding concentration indices are presented in Table 4. The results show that significant inequality is concentrated in FTs (CI 0.24, 95% CI 0.14/0.33, SE = 0.05). The concentration indices for MT (CI 0.02, 95% CI = 0.03/0.08, SE = 0.03) and DMFT (CI = 0.02, 95% CI = 0.02/0.06, SE = 0.02) were small and close to zero, while the concentration for DT (CI = − 0.04, 95% CI = − 0.01/0.02, SE = 0.03) was not statistically significant.

**Table 4:** Concentration index of DMFT, DT, MT and FT

	CI	95% Confidence interval		SE
DMFT	0.02	0.02	0.06	0.02
DT	− 0.04	− 0.01	0.02	0.03
MT	0.02	0.03	0.08	0.03
FT	0.24	0.14	0.33	0.05

### Decomposition of concentration index

#### Bivariate analysis

Table 5 presents bivariate analysis results that explore the potential determinants of FT among the participants. The number of FTs was associated with the type of household, education level, consumption of desserts, time of last dental visit, use of toothpicks, reimbursement for dental treatment, urban employee basic medical insurance system, new-type rural medical collaboration system and household income.

**Table 5:** Factors associated with FT: bivariate associations

	FT		95% CI		*P*
	Mean	SE			
*Gender*					
Male	0.67	0.12	0.44	0.90	0.17
Female	1.07	0.28	0.52	1.61	
*Type of household*					
Non-agricultural family	1.27	0.17	0.93	1.61	0.01
Agricultural family	0.54	0.11	0.32	0.77	
*Age*					
35–39 years	1.02	0.16	0.71	1.34	0.47
65–74 years	0.71	0.24	0.25	1.18	
*Education*					
Low education level	0.34	0.05	0.23	0.44	0.04
Medium education level	0.86	0.24	0.38	1.33	
High education level	1.08	0.21	0.68	1.48	
*Consumption of desserts*					
No	0.59	0.12	0.36	0.82	0.04
Yes	1.08	0.21	0.68	1.48	
*Consumption of sugared beverages*					
No	0.93	0.22	0.51	1.35	0.25
Yes	0.60	0.11	0.38	0.82	
*Time of last dental visit*					
Never visited dentist	0.06	0.03	0.00	0.12	0.00
Visited dentist 1 year ago	0.87	0.15	0.57	1.17	
Visited dentist 6–12 months ago	2.03	0.49	1.06	2.99	
Visited dentist within last 6 months	1.75	0.39	1.00	2.51	
*Tooth brushing*					
No	0.00	0.00	0.00	0.00	
Yes	0.87	0.18	0.51	1.23	
*Toothpick use*					
No	0.58	0.12	0.33	0.82	0.01
Yes	0.97	0.22	0.54	1.39	
*Flossing*					
No	0.82	0.17	0.48	1.16	0.00
Yes	1.72	0.25	1.24	2.21	
*Reimbursement for dental treatment*					
No	0.82	0.17	0.49	1.15	0.00
Yes	2.04	0.30	1.45	2.62	
*Urban employee basic medical insurance system*					
No	0.58	0.12	0.34	0.82	0.00
Yes	1.32	0.19	0.95	1.69	
*Urban residents medical insurance system*					
No	0.85	0.19	0.48	1.21	0.76
Yes	1.01	0.20	0.63	1.40	
*The new-type rural medical collaboration system*					
No	1.23	0.14	0.95	1.52	0.00
Yes	0.45	0.12	0.21	0.69	
*Self-rated oral health*					
Self-rate oral health as low	0.85	0.24	0.37	1.33	0.43
Self-rate oral health as Medium	0.98	0.22	0.56	1.41	
Self-rate oral health as High	0.64	0.08	0.47	0.80	
*Oral health knowledge*					
Low scores on oral health knowledge	0.79	0.18	0.44	1.14	0.17
High scores on oral health knowledge	1.17	0.29	0.60	1.75	
*Oral health attitude*					
Low scores on oral health attitude	0.50	0.08	0.34	0.66	0.17
High scores on oral health attitude	0.95	0.21	0.55	1.36	
*Chronic disease*					
No	0.85	0.14	0.57	1.12	0.70
Yes	0.92	0.32	0.29	1.55	
*Household income*					
Low	0.37	0.20	− 0.02	0.77	0.02
Medium	0.77	0.25	0.29	1.26	
Medium–high	0.85	0.22	0.41	1.28	
High	1.14	0.17	0.80	1.47	

#### Multivariate analysis

Table 6 presents the β value from the negative binomial model exploring the determinants of the number of FTs among the study participants.

**Table 6:** Multivariate analysis of determinants of FTs

Variable	β	*P* value	95% CI	
*Gender*				
Male	Base category			
Female	0.30	0.03	− 0.13	0.73
*Type of household*				
Non-agricultural family	Base category			
Agricultural family	− 0.53	0.02	− 0.99	− 0.08
*Age*				
35–44 years	Base category			
65–74 years	− 0.73	0.00	− 1.09	− 0.38
*Education*				
Low education level	Base category			
Medium education level	0.51	0.16	− 0.30	1.32
High education level	0.25	0.55	− 0.59	1.10
*Consumption of desserts*				
No	Base category			
Yes	0.03	0.36	− 0.29	0.36
*Consumption of sugared beverages*				
No	Base category			
Yes	− 0.26	0.19	− 0.73	0.21
*Time of last dental visit*				
Never visit dentist	Base category			
Visited dentist 1 year ago	2.70	0.00	1.88	3.51
Visited dentist 6–12 months ago	3.46	0.00	2.59	4.33
Visited dentist within last 6 months	3.18	0.00	2.42	3.93
*Toothpick use*				
No	Base category			
Yes	0.75	0.00	0.55	0.94
*Flossing*				
No	Base category			
Yes	0.38	0.41	− 0.28	1.05
*Reimbursement for dental treatment*				
No	Base category			
Yes	− 0.08	0.64	− 0.61	0.46
*Urban employee basic medical insurance system*				
No	Base category			
Yes	− 0.45	0.13	− 1.03	0.13
*Urban resident medical insurance system*				
No	Base category			
Yes	− 0.78	0.01	− 1.21	− 0.35
*The new-type rural medical collaboration system*				
No	Base category			
Yes	− 0.91	0.18	− 1.68	− 0.14
*Self-rated oral health*				
Self-rate oral health as low	Base category			
Self-rate oral health as Medium	0.00	0.51	− 0.32	0.32
Self-rate oral health as High	− 0.29	0.46	− 0.52	− 0.07
*Oral health knowledge*				
Low scores on oral health knowledge	Base category			
High scores on oral health knowledge	− 0.20	0.46	− 0.85	0.45
*Oral health attitude*				
Low scores on oral health attitude	Base category			
High scores on oral health attitude	0.25	0.10	− 0.09	0.60
*Chronic disease*				
No	Base category			
Yes	0.31	0.10	− 0.06	0.67
*Log household income*	0.04	0.04	− 0.28	0.35

The results from Table 6 highlight several significant predictors of the number of FTs among the study participants. In particular, household income, gender, type of household, age, use of toothpicks, time of last- dental visit and urban resident medical insurance system were positively associated with the number of FTs. In separate univariate analyses, variables including education, consumption of desserts, reimbursement for dental treatment, urban employee basic medical insurance system, and the new-type rural medical collaboration system were significant; however, they did not remain so in the multivariate analysis shown in Table 6. This is likely because of collinearity between variables. Despite this, we felt that the inclusion of all these variables in the multivariate model was important to capture the full effect of socioeconomic status on FT inequality.

#### Decomposition analysis of concentration index

Table 7 presents the results of the decomposition analysis of the variables contributing to FT inequality. The results are presented as the contribution and percentage contribution of each variable to the overall inequality in the concentration index. A positive contribution means that the variable increases the inequality of FT and vice versa. For socioeconomic-related FT inequalities, high-income people receive more dental fillings or low-income people receive fewer dental fillings, which can promote the inequality of FTs, and the contribution of the variable will be positive. However, only the number of low-income people who received fewer dental fillings should be rectified to reduce inequality. Therefore, we should combine the sign of the β value from the negative binomial model (Table 6) when we look at the contribution (Table 7) of certain variables.

**Table 7:** Concentration index and decomposition analyses for the number of FTs

Concentration index	0.24***	100.0%
Projected concentration index	0.26	110.9%
Residual term	− 0.03	− 10.9%

Variable	Contributions	Percentage contribution	Concentration index	Elasticises
Gender	0.00	0.1%	0.00	0.17
Type of household	0.02	7.4%	− 0.06	− 0.31
Age	0.00	− 1.1%	0.01	− 0.42
Education overall	0.01	2.2%	–	–
Low education level	Base	Base	–	–
Medium education level	− 0.02	− 10.2%	− 0.08	0.31
High education level	0.03	12.4%	0.29	0.10
Consumption of desserts	0.00	1.2%	0.13	0.02
Consumption of sugared beverages	− 0.01	− 2.4%	0.13	− 0.05
Last time dental attendance overall	0.23	96.8%	–	–
Never visited dentist	Base	Base	–	–
Visited dentist 1 year ago	0.06	24.9%	0.04	1.55
Visited dentist 6–12 months ago	0.07	28.7%	0.14	0.48
Visited dentist within last 6 months	0.10	43.2%	0.22	0.48
Toothpick use	0.02	9.4%	0.04	0.62
Flossing	0.01	3.5%	0.27	0.03
Reimbursement for dental treatment	0.00	− 0.8%	0.50	0.00
Urban employee basic medical insurance system	− 0.09	− 39.6%	0.40	− 0.23
Urban resident medical insurance system	0.00	− 0.5%	0.01	− 0.12
The new-type rural medical collaboration system	0.08	34.5%	− 0.20	− 0.42
Self-rated oral health overall	− 0.01	− 2.1%	–	–
Self-rate oral health as low	Base	Base	–	–
Self-rate oral health as medium	0.00	0.0%	0.07	0.00
Self-rate oral health as high	− 0.01	− 2.1%	0.06	− 0.08
High scores of oral health knowledge	− 0.02	− 6.5%	0.30	− 0.05
High scores of oral health attitude	0.00	0.7%	0.01	0.24
Chronic disease	0.01	3.0%	0.07	0.11
Log household income	0.01	5.1%	0.45	0.03

The significant for Concentration index was set at 0.05, *P < 0.05, **P < 0.01, ***P < 0.001

**Fig. 2:**
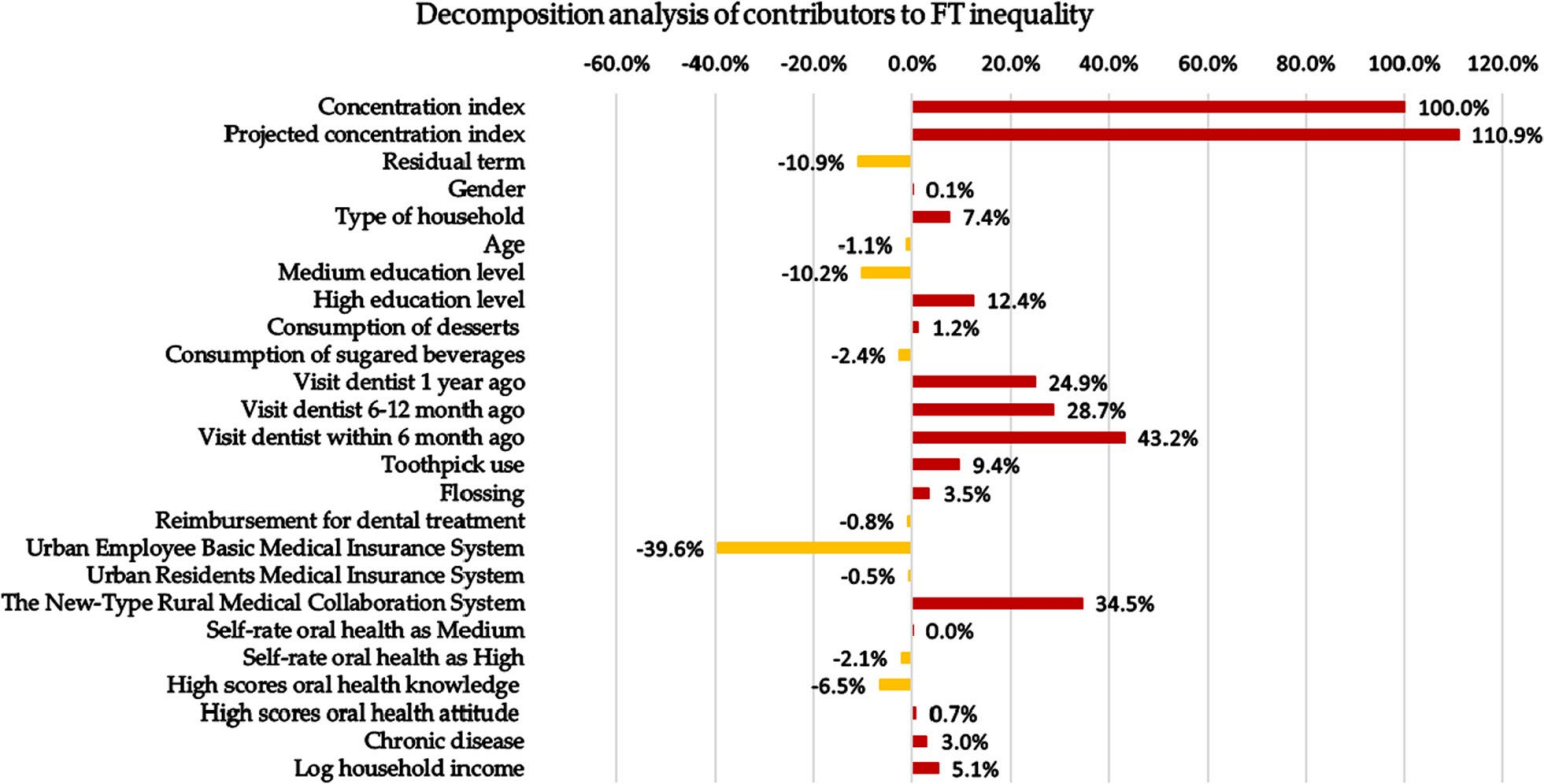
Decomposition analysis of contribution to FT inequality. Graphical representation of CI and decomposition analysis: positive contributions (right column) and negative contributions to inequality (left column)

Household income is positively associated with the FT number and contributes a large percentage to FT inequalities (5.1%). People with a high education level are more likely to access treatment for dental fillings and increase FT inequality (12.4%). Time of last dental visit also exacerbates FT inequality (96.8% in total). The new-type rural medical collaboration system (34.5%) contributes a positive percentage to FT inequality, while the urban employee basic medical insurance system (-39.6%) reduces FT inequality. Factors such as gender, type of household, consumption of desserts and sugared beverages, floss, toothpicks, oral health knowledge and attitudes explain only a small percentage of the inequality. Figure 2 presents the analysis in graphical form.

## Discussion

This study provides systematic information about dental caries and strong evidence of large socioeconomic-related oral health inequalities among adults in Guangdong Province. This study presents, for the first time, concentration indices for dental caries for adults in Guangdong Province. The findings indicate that oral health inequalities are more apparent in measures that reflect disease management than they are in outcome measures of the disease experience. The analysis shows us that decomposing socioeconomic inequalities into their specific determinants facilitates a more in-depth analysis of dental caries status in adults of Guangdong Province.

The high prevalence rates of caries evaluated by the DMFT, DT, MT, FT in the study were similar to those reported in Uganda [36], New Delhi [37] and a 2005 survey of China [38]. The high rates of MTs and DTs show that adults in Guangdong Province have poor oral health. A low number of FTs indicates a high rate of untreated DTs. Consistent with the poor oral health condition of these participants, this epidemiological survey found a lack of general oral health care behaviour. For example, only 5.0% of participants reported using floss daily. Moreover, 35.1% of participants had never visited a dentist, which explains the low number of FTs. The results remind us of the importance of effective oral health care, such as flossing and regular oral health examinations. In summary, there is still many opportunities for improvement in regard to oral health behaviours among adults in this region of China.

Social inequalities in a wide range of oral health parameters, both clinical and self-reported, have been documented. For example, some studies have found poorer self-rated oral health reported by individuals with low income and low education [3]. Wamala and colleagues reported that increased levels of socioeconomic disadvantage are associated with decreased use of dental services and poorer oral health among Swedish adults [39]. Household income and educational level are significantly associated with periodontitis and edentate status in elderly people [40, 41]. Our findings support the standpoint of the previous study. The CI of FTs was particularly prominent compared with those of the DMFT, DT and MT, which indicated greater inequalities in dental caries treatment than in caries experience in Guangdong Province. The lower social status did not necessarily translate into higher disease experience (i.e., DMFT); however, it was associated with the nature of dental treatment reported to have been received in the past year. The low rates of dental visits among the study participants may explain the phenomenon. Therefore, determining specific determinants leading to dental treatment inequalities and providing greater access to dental care for lower-income populations is necessary.

Factors such as household income and high education level increase inequalities and play a large role in explaining inequalities in the number of FTs among adults in Guangdong Province. This finding was similar to that of a previous study that reported social gradients with respect to untreated dental disease in both children and adults [7]. It is interesting to note that compared with a low education level, a high education level increases the inequality of FTs, while a medium education level reduces the inequality. Further analysis shows that this is because the concentration index of the medium education level tends to be concentrated in low-income groups (concentration index = − 0.08), and the low-income groups with medium education likely obtain more dental fillings. This finding indicates that universal basic compulsory education seems to promote equality in regard to dental caries filling. Education level is a predictor of health outcomes and influences individual and social levels [42]. It is possible that adults with basic compulsory education or higher education levels have greater awareness of self-health, access to resources, and understanding of how to navigate the health care system [43]. The analysis reports associations at one point in time; however, the issues of access, use, and treatment are repeated in the life course, leading to inequalities in oral health outcomes later in life. Therefore, the inequality of oral health resources is an urgent problem to be addressed. This finding reminds us of the importance of improving the overall national economy and education level to enable low-income groups to obtain more and accessible oral health care resources.

To reconstruct the public medical insurance system and widen insurance coverage to universal coverage, the Chinese government developed the following medical safeguard systems for its citizens: the urban employee basic medical insurance for the urban employed, which was initiated in 1998; the new-type rural collaboration medical system for rural residents, which was established in 2003; and the urban resident basic medical insurance, which was piloted in 2007 and covers urban residents without formal employment [44]. Additionally, China achieved universal health coverage for over 95% of its population in 2011 with benefits that include prevention and comprehensive treatment services [45]. In this study, the new-type rural medical collaboration system contributed a positive percentage to FT inequality. The results indicated that people in the new-type rural medical collaboration system (low-income group) were not eligible to access necessary oral treatment, which widened the FT gap between people with or without the new-type rural medical collaboration system. Urban employee basic medical insurance contributed a negative percentage to FT inequality. Thus, it seemed that urban employee basic medical insurance was a measure to reduce inequality. However, further analysis found that urban employee basic medical insurance was negatively associated with the number of FTs, which means that it reduced inequality because people with that insurance (high-income group) are also less likely to access dental care treatment. The findings indicated that, at present, all medical safeguard systems in China fail to encourage people to use medical resources and reduce income-based disparities in oral health. A previous study [46] also reported that oral health care in China is delivered by a large government-controlled public sector, with over 85% of the total expenses covered by patients' out-of-pocket payments. In recent years, the numbers of dentists and oral health service providers have increased, although oral health services are not being utilized efficiently [47]. The study indicated that the Chinese health care insurance system is intended to improve access to affordable health care for all and to alleviate inequalities in access to care that exist among rural residents and low-income households.

The current study had several limitations. First, oral health outcomes and relevant inequalities are caused by multiple factors. However, the current study discussed only some of these factors. Second, reporting bias existed in questionnaires because of the different levels of comprehension among participants. Therefore, our results should be interpreted considering these limitations.

The strengths of the current study are as follows. First, the analyses distinguished between the occurrence and the management of dental caries, and it found that dental caries inequality centres on FTs. Second, the study used the concentration index and decomposition analysis to establish the determining factors associated with the inequality of FTs. Finally, given the national debates about dental caries inequality, these results can provide a basis for discussion about measures to reduce oral health inequalities. Future research about reducing oral health inequality should focus on how to implement appropriate, targeted education programs directed at all socioeconomic groups, regardless of wealth and education. Meanwhile, health care reform should aim to improve access to affordable health care for all residents, especially rural residents and low-income households.

## Conclusions

The study findings indicate that oral health inequalities may centre on dental disease management as opposed to dental disease experience and that a lower social position may not necessarily translate into a higher disease experience.

The results of the decomposition analysis of FT inequality indicated that among the studied factors, household income, education level, and regular oral health examinations have the greatest contribution to the inequalities in the number of FTs. More importantly, the current medical insurance systems are not being effectively used in oral treatment. Therefore, health care reform should focus on enhancing subsidies for the lower-income groups to improve the treatment rates and narrow the inequality of oral health. These conclusions may be very useful in policy-making and in the implementation of interventions for tackling socioeconomic inequality in the number of FTs.

## Data Availability

The datasets used and analysed during the current study are available from the corresponding author upon reasonable request.

## Abbreviations

CC: Concentration curve
CI: Concentration index
95% CI: 95% Confidence interval
SE: Standard error
DMFT: Decayed, missing and filled teeth
DT: Decayed teeth
MT: Missing teeth
FT: Filled teeth
